# Improving 3D Reconstruction Through RGB-D Sensor Noise Modeling

**DOI:** 10.3390/s25030950

**Published:** 2025-02-05

**Authors:** Fahira Afzal Maken, Sundaram Muthu, Chuong Nguyen, Changming Sun, Jinguang Tong, Shan Wang, Russell Tsuchida, David Howard, Simon Dunstall, Lars Petersson

**Affiliations:** 1 Data61, Commonwealth Scientific and Industrial Research Organisation (CSIRO), Canberra, ACT 2601, Australia; sundaram.muthu@data61.csiro.au (S.M.); chuong.nguyen@data61.csiro.au (C.N.); changming.sun@data61.csiro.au (C.S.); jinguang.tong@anu.edu.au (J.T.); shan.wang@anu.edu.au (S.W.); russell.tsuchida@data61.csiro.au (R.T.); david.howard@data61.csiro.au (D.H.); simon.dunstall@data61.csiro.au (S.D.); lars.petersson@data61.csiro.au (L.P.); 2 School of Computing, Australian National University (ANU), Canberra, ACT 2601, Australia

**Keywords:** sensor noise modeling, 3D reconstruction, RGB-D fusion

## Abstract

High-resolution RGB-D sensors are widely used in computer vision, manufacturing, and robotics. The depth maps from these sensors have inherently high measurement uncertainty that includes both systematic and non-systematic noise. These noisy depth estimates degrade the quality of scans, resulting in less accurate 3D reconstruction, making them unsuitable for some high-precision applications. In this paper, we focus on quantifying the uncertainty in the depth maps of high-resolution RGB-D sensors for the purpose of improving 3D reconstruction accuracy. To this end, we estimate the noise model for a recent high-precision RGB-D structured light sensor called Zivid when mounted on a robot arm. Our proposed noise model takes into account the measurement distance and angle between the sensor and the measured surface. We additionally analyze the effect of background light, exposure time, and the number of captures on the quality of the depth maps obtained. Our noise model seamlessly integrates with well-known classical and modern neural rendering-based algorithms, from KinectFusion to Point-SLAM methods using bilinear interpolation as well as 3D analytical functions. We collect a high-resolution RGB-D dataset and apply our noise model to improve tracking and produce higher-resolution 3D models.

## 1. Introduction

Depth sensing is an essential component in perceiving the environment in three dimensions. With depth sensors providing rich 3D information, they have become integral to vision systems across manufacturing and other industries, facilitating various applications including 3D object segmentation, pose estimation [1], 3D reconstruction, and scene understanding [2]. Depth sensors enable the creation of accurate 3D models for printing, surface inspection, or part repair in reverse modeling. Specifically, advanced digital manufacturing applications including metrology, quality inspection, autonomous repair, 3D printing, and others require high-resolution scanning with low measurement uncertainty and submillimeter-level accuracy in the 3D reconstruction of the scanned objects. This is essential to capture fine details, achieve tight tolerances, meet dimensional specifications, or achieve the required surface finishing.

However, none of the raw depth values from the current depth sensors on the market are capable of achieving reliable submillimeter accuracy in the 3D reconstruction of scanned objects required for high-precision manufacturing applications. This limitation stems from noisy depth maps, characterized by high measurement uncertainty, which is the result of a combination of systematic errors (inherent to the system), and non-systematic errors (caused by uncontrolled environmental factors).

Systematic errors originate from the sensing methodology involved, such as structured light, time of flight, or stereo depth sensors [3]. Sensor calibration is typically performed to reduce the effect of systematic errors to some extent. Non-systematic errors originate from environmental influences such as the lighting conditions, the reflectivity of the target objects, measurement distance, and the angle between the object’s surface normals and the principal axis of the camera. To this end, approaches in [3,4,5,6,7,8] have empirically modeled the noise characteristics of well-known low-cost RGB-D sensors such as Microsoft Kinect and Intel RealSense.

Zivid is a newer high-resolution 3D structured light camera whose noise characteristics have not yet been extensively studied. We focus on high-precision 3D scanning for manufacturing inspection applications, and this paper aims to model the noise characteristics of the Zivid sensor to optimize its utility in such applications. We quantify the noise in terms of axial noise (in the direction of the principal axis of the camera) and lateral noise (in the directions perpendicular to the axis of the camera), examining how these components vary with object depth and surface angle. Additionally, our study evaluates the influence of lighting conditions, exposure time, and the number of captures on the quality of depth maps obtained with the Zivid sensor (Zivid, Oslo, Norway).

**Figure 1:**
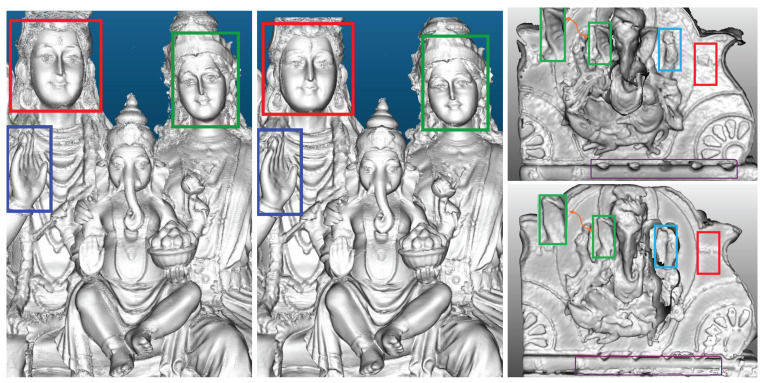
Our noise model, when integrated with KinectFusion [10], improves the quality of the surface estimation, highlighted in colored boxes (**middle**) compared with the quality without noise filtering (**left**). On the right, the noisy depth filtering using our noise model (**top right**) effectively captures high-resolution details, such as the ridges in the ear (green box) and the background texture (red box), which are absent in the unfiltered version (**bottom right**).

In order to illustrate the use of our noise modeling to improve downstream tasks, we integrate our noise model with traditional methods such as KinectFusion and with modern neural-based approaches like Point-SLAM [9]. Filtering noisy depth values using our noise model not only improves the quality of 3D reconstruction but also captures high-resolution details, as demonstrated in Figure 1.

   There is a notable absence of high-precision benchmark RGB-D datasets for submillimeter-level 3D reconstruction. To fill this gap, we introduce a new dataset captured with the Zivid camera mounted on a robotic arm. This dataset features objects with complex geometry, scanned from multiple viewpoints achieved by rotating both the object on a turntable and the camera on a robot arm with high precision.

The main contributions of this paper are as follows:

- To improve 3D scanning in advanced manufacturing, we empirically model the axial and lateral noise characteristics of a high-resolution RGB-D Zivid sensor as a function of the measurement distance and surface angle of the scanned objects. We also provide insights on performance under different lighting conditions, exposure time, and different capture settings.
- We demonstrate how to employ our noise models in 3D reconstruction pipelines from traditional to neural-based methods to improve both the quality of 3D reconstruction and pose estimation using bilinear interpolation as well through fitted analytical functions.
- We collect and publish a new dataset that contains high-resolution RGB-D scans of objects with complex geometry that can be used in many applications including pose estimation and 3D reconstruction.

## 2. Related Work

In the following, we briefly review various noise characterization methods and their applications to improve computer vision tasks. We then provide an overview of the 3D reconstruction methods using RGB-D sensor data.

### 2.1. Noise Modeling of RGB-D Sensors

Systematic and non-systematic errors generally influence the accuracy of depth measurements from RGB-D sensors. The first detailed study of the noise characteristics of the well-known Kinect V1 RGB-D sensors was performed in [8]. In particular, the authors performed a calibration step to model the systematic errors and derived a theoretical random noise model to account for non-systematic errors. Nguyen et al. [5] present an empirical model for measuring the lateral and axial noise distributions of the Kinect V1 sensor related to the distance and angle from the surface. Similarly to Nguyen et al. [5], Choo et al. [11] characterize the noise model of the Kinect sensor in both the axial and lateral directions. In addition, Fankhauser et al. [6] characterize the influence of ambient light for indoor, overcast, and direct sunlight situations, considering the angle of incidence of sunlight. Tölgyessy et al. [4] extend the analysis to include the Azure Kinect, examining its noise characteristics during warming up, reflectivity levels, pixel positions, and in outdoor lighting conditions.

With the growing variety of RGB-D sensors in the market, several studies [3,7,12] compare the noise characteristics of different RGB-D sensors, including various versions of the Kinect. Mallick et al. [7] provide a comprehensive review of the methods that characterize the Kinect sensor noise. Additionally, they introduce and measure interference noise when multiple Kinect sensors are used. Jing et al. [3] compare the depth image quality and noise characteristics of the Kinect V1, Kinect V2, and the PrimeSense sensors. Halmetschlager et al. [12] compare the noise characteristics of ten different RGB-D sensors. They suggest employing structured light cameras for robots operating in uncontrolled conditions due to the comparatively lower noise variation compared with other sensor technologies.

While the previously mentioned methods rely on a calibration target object for noise characterization, some approaches [13,14,15] estimate noise or depth uncertainty directly from the captured data. Dryanovski et al. [15] propose a mixture-of-Gaussians convolution method to quantify the uncertainty in a single depth frame. Compared with the approaches in [5,8], Lu and Song [15] represent the uncertainty around the edges of the object more accurately. Proenca et al. [14] propose a depth filter that combines approaches in [8,15] to model the uncertainty of spatial and temporal depth measurement. Dong et al. [13] propose a regularization technique that models depth uncertainty in the incremental Bayesian update step to better handle outliers.

In contrast to these preceding contributions, our focus is on modeling the noise characteristics of the high-resolution Zivid camera. The Zivid camera is well-suited for advanced manufacturing applications that require more precise measurements, but empirical noise studies for this particular sensor are lacking in the existing literature.

### 2.2. Application of Noise Models to Improve Computer Vision Tasks

Noisy depth maps degrade the quality of the scans and reduce the accuracy of downstream applications such as pose estimation, point cloud alignment, SLAM, robot perception, and 3D reconstruction. Hence, various studies have utilized sensor noise models to filter or fuse depth maps to enhance the accuracy of these tasks [5,8,16,17,18,19,20,21,22,23,24,25].

For SLAM applications, Gutierrez et al. [16,17] improve the dense visual odometry method (DVO SLAM) [26,27] by considering depth errors. Specifically, Gutierrez-Gomez et al. [16] parameterize geometric error using inverse depth, while dense noise aware SLAM (DNA-SLAM) [17] considers noise explicitly and uses a sophisticated weighting mechanism to improve odometry. Yamaguchi et al. [18] integrate the noise characteristics of the Kinect sensor into the iterative closest point (ICP) algorithm, improving the accuracy of SLAM. Furthermore, feature-based SLAM methods [21] use depth uncertainty models derived from sensors such as structured light cameras to improve odometry despite their lower depth resolution. Bingham procrustean alignment [24] uses uncertainty estimates from noisy depth images to extract reliable surface features for robust pose alignment.

A robot perception system in [20] employs the sensor noise model [5] to achieve optimal mapping quality. Dhawale et al. [28] employ the sensor noise model [14] to accurately compute the correspondences between the existing map and the sensor observation during incremental probabilistic map updates. In addition, incorporating sensor noise characteristics reduces the memory requirement of the map representation. Voxblox [29] improves mapping and path planning in unknown environments for autonomous flight applications by using truncated signed distance fields based on the noise model (TSDF) [5]. Saulnier et al. [25] decrease the uncertainty of the map in the autonomous exploration task by taking into account the noise based on the measured depth.

In relation to 3D reconstruction and other applications, Nguyen et al. [5] demonstrate that exploiting the noise model in the KinectFusion system [10] improves pose estimation accuracy and allows reconstruction of finer details, smaller objects, and thinner surfaces. Iversen et al. [23] exploit a noise model [5] for simulating Kinect V1 depth images, which can be used in various computer vision algorithms requiring a substantial dataset. Recently, Ran et al. [30] have introduced an implicit neural representation for path planning and active reconstruction, using the sensor noise model to assess reconstruction uncertainty to determine the next best view for active scanning. Katar et al. [31] model lateral and axial noise in 3D camera data to enhance synthetic training datasets, demonstrating that this improves neural network performance in real-world object segmentation tasks. Similarly, Cai et al. [32] quantify and model non-systematic noise in the PMD Flexx2 Time-of-Flight depth camera, proposing Gaussian-based models for axial and lateral noise to aid in accurate sensor simulation for robotic applications. In Rustler et al. [33], the authors evaluate the performance of four stereoscopic RGB-D cameras—Intel RealSense D435, Intel RealSense D455, StereoLabs ZED 2, and Luxonis OAK-D Pro—across various scenarios, providing insights into their suitability for different robotic applications. The research in [34] assesses the performance of three depth sensors—Intel RealSense D415, Intel RealSense D405, and Stereolabs ZED-Mini—within close-range surgical simulation settings, highlighting their accuracy, fill rate, and suitability for reconstructing anatomical structures. Additionally, Keetha et al. [35] propose methods to enhance depth value approximation in 3D image-based modeling for complex objects by introducing noise filtering and inverse perspective mapping techniques, resulting in improved accuracy and processing speed.

### 2.3. 3D Reconstruction from Multi-View RGB-D Scans

Recent research in 3D reconstruction and scene understanding is broadly categorized into two distinct approaches. Initially, fusion-based methods were pioneered, drawing heavily on the foundational KinectFusion algorithm introduced by Izadi et al. [10]. This line of work was significantly advanced by Nießner et al. [36], who facilitated real-time, large-scale reconstruction through voxel hashing. Subsequently, DynamicFusion [37] expanded these capabilities to dynamic scenes via a novel warp field technique. MaskFusion [38] introduced an innovative approach to segment, track, and reconstruct moving objects by integrating semantic segmentation. BundleFusion [39] further refined these methodologies by proposing a comprehensive system that ensures real-time, globally consistent 3D reconstructions through on-the-fly optimization and surface re-integration. On the other hand, the exploration of neural radiance field (NeRF)-based methods represents a shift towards leveraging deep learning for scene reconstruction. GO-SLAM [40] employs an SDF-based NeRF for scene reconstruction, whereas Point-SLAM [9] utilizes Point-NeRF [41] for similar purposes. Our noise model improves 3D reconstruction accuracy in both fusion-based and neural network methods.

## 3. Noise Modeling

### 3.1. Noise Characteristics

**Figure 2:**
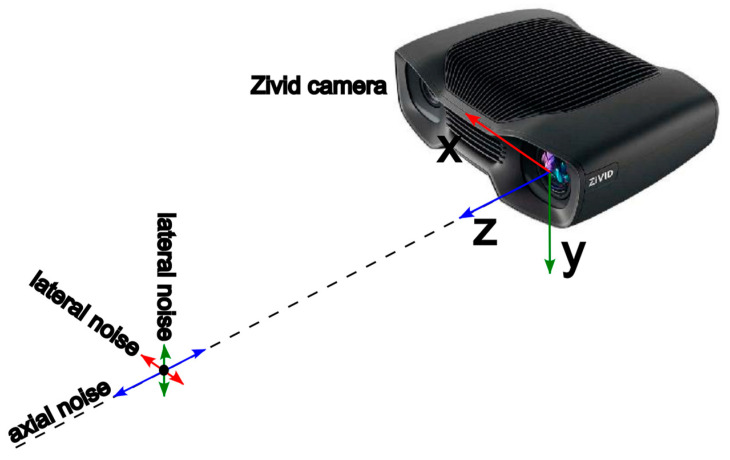
Illustration of Zivid camera noise components at an arbitrary point, *P(x, y, z)*, measured by the camera. Axial noise, $\sigma_{Z}$, and lateral noise, $\sigma_{L}$, represent the uncertainty of the measured location of point *P*, along with the z-axis and x- and y-axes, respectively.

Depth measurement errors in structured light sensors can occur due to sensor warm-up, and varying depth distances, angles, binning modes, reflectivity, and distortion. In this paper, we characterize the noise in three dimensions as a function of depth distance, *z*, and angles, θ, similar to [5]. Specifically, we quantify the noise characteristics in terms of axial noise, $\sigma_{Z}=f\left(z,\theta \right):R\times R\to R$, which denotes noise along the principal axis of the camera, and lateral noise, $\sigma_{L}=g\left(z,\theta \right):R\times R\to R$, representing noise in directions perpendicular to the camera axis, as shown in Figure 2.

### 3.2. Experimental Setup

**Figure 3:**
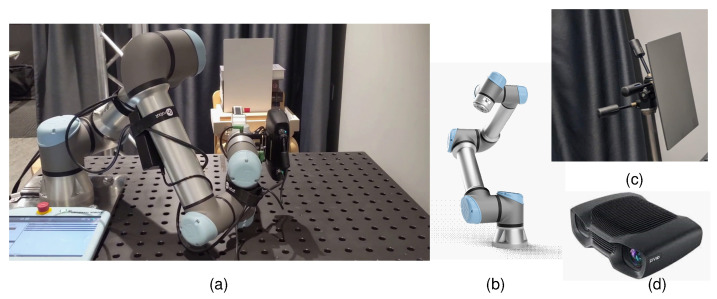
Our experimental setup for modeling sensor noise includes a robot arm (**b**), a planar target (**c**), and a Zivid 2 RGB-D structured light sensor (**d**), as shown in (**a**).

Figure 3a shows the experimental setup used to collect data to estimate the noise functions *f* and *g*. It consists of a robot arm, camera, and a planar target. We use the Zivid 2 (https://www.zivid.com/zivid-2, accessed on 20 January 2025) structured light RGB-D camera whose noise model is to be estimated, as shown in Figure 3d. We use a planar target (a calibration board (https://calib.io/, accessed on 20 January 2025) that has a dimensional accuracy of 10 μm) that is mounted on a tripod, as shown in Figure 3c. The planar target and the axis of rotation of the planar target are both perpendicular to the principal z-axis of the camera. Additionally, the camera is aligned to focus on the center of the plane. The target is capable of rotating freely around the vertical axis. The camera is mounted on the UR5e robot arm (https://www.universal-robots.com/products/ur5-robot/, accessed on 20 January 2025) to automate data collection precisely. An eye-in-hand calibration step is performed to determine the transformation between the camera and the robot base, ensuring accurate pose estimates of the Zivid camera within a global coordinate system. We vary the following parameters in our setup to collect data to model various noise characteristics of the sensor.

- *Distance z*: the distance between the target and the camera, z, is varied from 37.5 cm to 107.0 cm in steps of 2.5 cm. This axial movement of the camera is performed by moving the robot arm towards and away from the target in a straight line perpendicular to the planar target.
- *Angle θ*: the angle of the planar target is varied from 0° to 90° in steps of 10° with rotation perpendicular to the camera’s principal axis.
- *Lighting conditions*: the data collection process involves scans of the calibration board under both light and dark conditions at 0° for all distances.
- *Exposure time*: the exposure time or the duration of the shutter opening time is varied from 1677 ms to 100 s.
- *Number of captures*: the number of captures is varied from 1 to 5, combining multiple acquisitions with different apertures to enable capture at a high dynamic range.

### 3.3. Axial Noise

Axial noise refers to depth measurement errors that vary along the sensor’s viewing axis, characterized by deviation of points from a plane fitted to the scan of the planar target.

**Plane fitting:** we initially extract a clean surface of the calibration board from the scan, excluding boundaries to eliminate lateral noise effects and other unwanted effects such as specular reflections. Subsequently, a plane is fitted to the clean extracted surface and the error is estimated as a deviation between the plane fitted and the surface points. This process is repeated for all distances covered by the captured point clouds, across angles ranging from 0° to 70°.

Given a set of *N* points $P={\left\{p_{i}\right\}}_{i=1}^{N}$ from the extracted clean point cloud, where $p_{i}=\left(x_{i},y_{i},z_{i}\right)\in R^{3}$, at a distance *z* and angle θ with respect to the Zivid camera, the plane fitting problem aims to find the coefficients X={a,b,c,d}, such that the plane equation ax+by+cz+d=0 best fits the given points in the least square sense.

**Figure 4:**
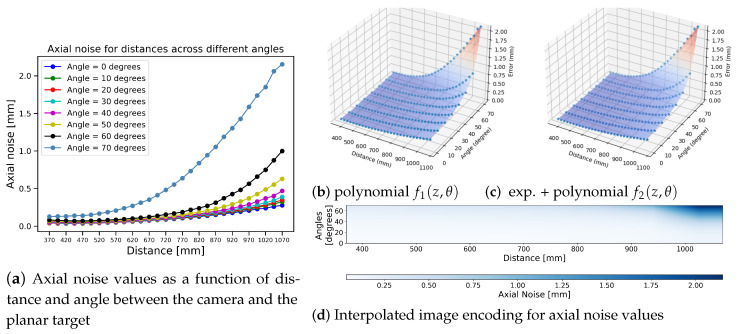
Axial noise modeling. (**b**,**c**) Fitted surface models for axial noise with a (**b**) 7th order bivariate polynomial, (**c**) bivariate exponential function plus a 2nd order bivariate polynomial, and (**d**) bilinearly interpolated image encoding for axial noise values corresponding to (**a**). The axis labels and titles are provided for illustrative purposes.

**Fitted axial noise model**: the axial noise for a single scan can then be calculated as the root mean square distance between the fitted plane coefficients and the points. Figure 4a shows axial noise values as a function of depth and angles. The axial noise is more pronounced at larger distances for an angle of 50° or greater. The noise exhibits a non-linear trend, increasing as both the surface distance and surface angle increase.

To capture this non-linear trend, we apply three distinct methods to fit a function to the noise curves in Figure 4a, and we select the one most convenient for simplified integration into 3D reconstruction algorithms.

**Polynomial fitting:** we fit surface models for the axial noise data displayed in Figure 4a. The model used in Figure 4b is represented as $f_{1}\left(z,\theta \right)=\sum_{i=0}^{7}\sum_{j=0}^{7-i}a_{ij}z^{i}\theta^{j}$ for an order 7 bivariate polynomial, with 36 coefficients on $a_{ij}$.

**Polynomial+exponential fitting:** to reduce the number of parameters fitted, we choose to mix both polynomials and exponential terms in the fitting to handle distortions and distance variations. The model used in Figure 4c is $f_{2}\left(z,\theta \right)=Se^{Az^{2}+Bz\theta +C\theta^{2}+Dz+E\theta }+az^{2}+bz\theta +c\theta^{2}+dz+e\theta +f$, with {S,A,B,C,D,E,a,b,c,d,e,f} being the coefficients. There are still 12 coefficients for $f_{2}\left(z,\theta \right)$. The values of all coefficients for both models are provided in the supplementary material in Tables S1 and S2.

**Look-up image:** due to the complexity of higher-order coefficients in both fitted surfaces, we generate an alternate look-up image, as shown in Figure 4d, whose pixels store values of the axial noise obtained in Figure 4a. Using bilinear interpolation, noise values are interpolated for any combination of distance and surface angle. This resulting axial noise image can easily be queried in downstream applications, such as KinectFusion [10], to obtain the noise value corresponding to a given distance and angle to achieve better 3D reconstruction. Table S3 in the Supplementary Material lists the axial noise values corresponding to distance and angle as shown in Figure 4.

### 3.4. Lateral Noise

Lateral noise refers to depth measurement errors that occur along the directions perpendicular to the camera axis that mainly looks at edge pixels of the planar target scan.

**Figure 5:**
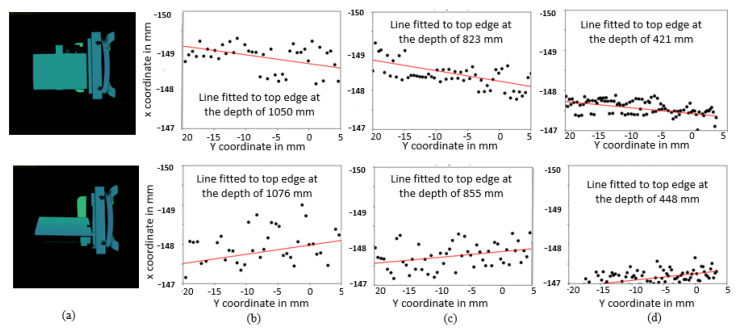
Pre-processing steps for lateral noise modeling. (**a**) Depth map with segmented edge of the planar target (marked in red color); Line fitted to pixels corresponding to the edge: at *far* distance (**b**), at *medium* distance (**c**), and at *near* distance (**d**). Rows indicate the angle of rotation of target, varying from 0° (**top**) to 60° (**bottom**).

**Line fitting:** Figure 5 explains the steps involved in lateral noise modeling from depth maps. Firstly, we remove the background pixels of the planar target. Then, we find the position of the top edge pixels of the planar target in the depth map. We limit the number of pixels to the middle 200 points of the top edge to avoid the effects of optical distortion. Subsequently, we perform a line fitting on the *x* and *y* coordinates of the extracted edge pixel points. We then calculate $\sigma_{L}$ as the root mean square distances between the pixel coordinates of the edge and the fitted line.

**Figure 6:**
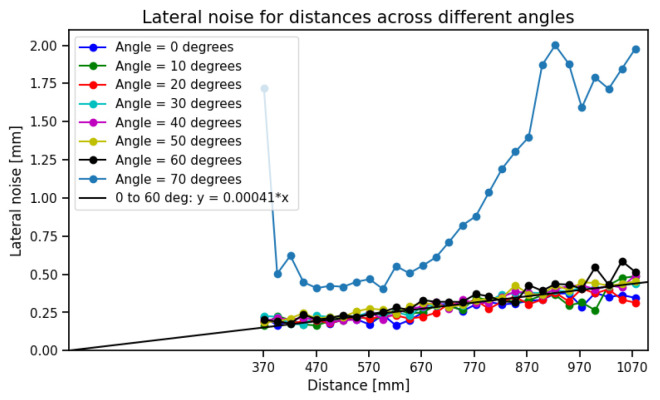
Lateral noise against different distances and angles.

**Fitted lateral noise model:** this procedure is repeated for all scans obtained across distances and angles ranging from 0° to 80°. Ideally, a single line should adequately represent all edge pixel coordinates, but due to lateral noise effects this is not always the case. Figure 5 shows that the spread of the points around the fitted line decreases with decreasing distances. We observe that the lateral noise is independent of the surface angles from 0° to 60°, but increases significantly for 70° to 80°, indicating the reduced reliability of the measurements at extreme surface angles. Figure 6 shows the fit of the linear model to the lateral noise, $\sigma_{L}$, as a function of surface distance.

### 3.5. Other Effects to Sensor Noise

**Figure 7:**
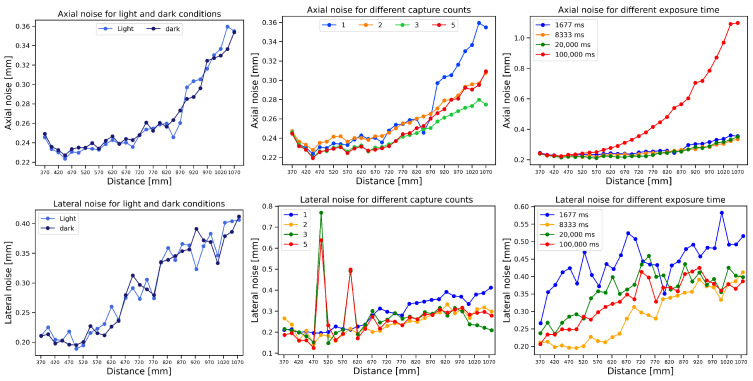
Variations in axial (**top**) and lateral noise (**bottom**) due to lighting conditions (**left**), number of captures (**middle**), and exposure time (**right**) as a function of distances at fixed 0°.

In this section, we explore the impact of varying lighting conditions, exposure times, and capture settings on axial and lateral noise as functions of distance. Figure 7 (left) shows that both axial and lateral noise are independent of the background lighting, since the light emitted by the structured light sensor is much stronger compared with the ambient background lighting. Figure 7 (middle) shows that the quality of the scans can be improved by increasing the number of captures, each with different aperture settings, to capture with a high dynamic range. Figure 7 (right) shows that increased exposure time leads to a decrease in lateral noise due to the smoothing effect at the depth edges. However, for axial noise, the quality of the depth images becomes degraded for extended exposure time due to sensor saturation.

## 4. Applying Noise Model to Improve 3D Reconstruction

To validate the effectiveness of our noise model, we capture a dataset using a camera mounted on a robot arm, with objects placed on a turntable. We then integrate our noise model into both traditional and implicit neural-based 3D reconstruction methods to improve the quality of both 3D reconstruction and pose estimation. We primarily seek to demonstrate how our modeled noise improves the quality of reconstruction using traditional KinectFusion, which is known for its reliability, compared with modern neural-based reconstruction methods [9].

### 4.1. Datasets

**Figure 8:**
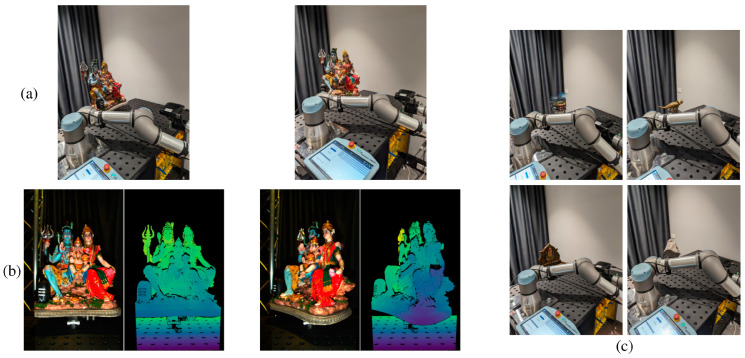
The experimental setup for dataset collection. From top to bottom: (**a**) complete setup with a robot arm, camera, and object placed in a rotating table for 0° and 45° rotation of the object, (**b**) corresponding RGB and scaled depth images captured, and (**c**) other object sequences captured—Dragon, Ganesh, Rock, and Dino.

In our experiment, we scan a variety of test objects mounted on a motorized turntable, using a Zivid camera integrated with a UR5e robotic arm. The test objects are cultural artifacts (Shiva and Ganesh), toy models (Dino and Dragon), and precision-engineered components (Gripper and Controller), as depicted in Figure 8c. The scanning process involves rotating each object on the turntable with an incremental step of 1°. At each step, the Zivid camera captures an RGB-D image, resulting in a total of 360 images per object along a circular path. This precise rotational increment facilitates the effectiveness of camera tracking algorithms, which often operate under the assumption of small angular changes. Consequently, the generated dataset provides comprehensive ground-truth information, enabling quantitative comparisons for each scanned object.

**Figure 9:**
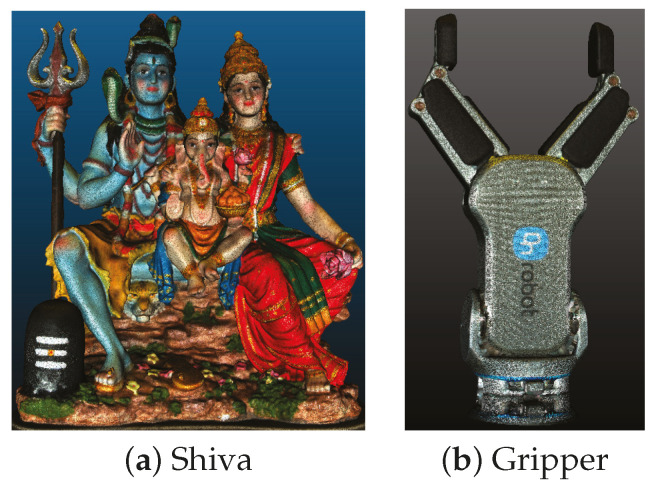
Poisson 3D surface reconstructions with point cloud data merged based on pair-wise ICP registration followed by pose graph optimization for (**a**) Shiva and (**b**) Gripper.

In order to obtain a baseline for 3D reconstruction for the dataset, we use traditional ICP registration for pose estimation and fusing multiple point clouds, followed by Poisson reconstruction for generating a mesh. Point-cloud data are filtered using a Euclidean cluster extraction algorithm, and then pair-wise data registration is performed using ICP to obtain the transformation parameters between two consecutive camera poses, followed by pose graph optimization. A subset of the whole point cloud data is chosen for merging to reduce the data size. Finally, Poisson 3D surface reconstructions are performed on the data obtained. Figure 9 shows the 3D surface reconstructions obtained for the Shiva and Gripper datasets.

### 4.2. Improving KinectFusion Using Our Noise Model

Following a similar approach as [5], we integrate our noise model into the KinectFusion algorithm [10] to improve the approximation of the truncated sign distance function (TSDF) for each voxel on the voxel grid. KinectFusion iteratively fuses depth data from an RGB-D camera to a voxel grid structure stored on the GPU. Within this grid, surface details are implicitly encoded as signed distances, truncated to a predefined layer containing the expected surface of an object. The integration of new depth values into the voxel grid employs a weighted average scheme. The global pose of the camera is tracked using a linearized ICP algorithm (using small angle assumption) [42] by aligning the current frame with the accumulated model. The implicit surface can be extracted by either ray-casting the voxel grid or triangulating a mesh using marching cubes. A 2D pixel coordinate on the depth map is denoted as u=(x,y). $D_{i}\left(u\right)$ is the depth value at pixel *u* retrieved at the *i*th frame. With an intrinsic calibration matrix, *K*, which is typically a 3×3 matrix containing the camera’s focal lengths and optical center, a 3D vertex corresponding to the pixel *u* is $v_{i}\left(u\right)=D_{i}\left(u\right)K^{-1}\left[u,1\right]$. Here, $K^{-1}$ is the inverse of the intrinsic matrix *K*, and [u,1] is the homogeneous coordinate representation of the pixel *u*. $D_{i}$ therefore produces a single vertex map, $V_{i}$, which represents the 3D points for all pixels at the *i*th frame. The camera pose in the *i*th frame is represented as $T_{i}=\left[R_{i},c_{i}\right]$, where $R_{i}$ is the rotation matrix and $c_{i}$ is the translation vector, which describes the position and orientation of the camera relative to the world coordinate system. The position of the vertex in the global coordinates is then expressed as $v_{g_{i}}=T_{i}\;\cdot \;v_{i}$, where $v_{g_{i}}$ is the transformed vertex in the global coordinates, and $T_{i}\;\cdot \;v_{i}$ applies the rotation and translation to the 3D point $v_{i}$. The normal vector $n_{i}\left(p_{k}\right)$ at a point $p_{k}$ in 3D space represents the direction perpendicular to the surface at that point. It is used to calculate the angle, θ, between the z-axis and the normal, allowing surface data to be weighted based on the orientation of the depth measurement, with smaller angles indicating more reliable contributions to the updated TSDF.

Algorithm 1 states the KinectFusion algorithm in which our noise model has been incorporated for enhancing the TSDF values leading to improved depth fusion. The TSDF calculation at line 26 of the algorithm is designed to generate values within the range of [−1, 1]. In contrast to [10], where a uniform weight, *w*, is assigned to all depth measurements regardless of their noise levels for reconstruction updates, we address this issue by assigning weights to depth measurements based on their noise quality and distance from the camera, as shown in Line 27 of Algorithm 1. The exponential term represents the weight of the noise distribution. $\sigma_{z}\left(z_{\min}\right)$ represents the axial noise value at the minimum depth ($z_{min}$) used in the noise modeling process. For a detailed explanation of the steps of the KinectFusion [10] algorithm, refer to [5] and the references therein.
**Algorithm 1** TSDF integration considering both axial and lateral noise using bilinear interpolation  1:**Input:**  2: Depth map $D_{i}$ for frame *i*  3: Camera pose $T_{i}$ consisting of rotation matrix $R_{i}$ and translation vector $c_{i}$  4: Noise models: $\sigma_{Z}$, $\sigma_{L}$  5: Angle threshold $\theta_{max}$  6: Size, location and resolution of 3D volume *V* containing the object to be reconstructed  7: **Output:**  8: Updated TSDF $tsdf_{i}$ for the current frame  9:**for** each voxel *g* on *x*-*y* slice of volume *V* in parallel **do**10:    **while** sweep along *z*-axis of volume **do**11:        $v_{g}\leftarrow$ convert *g* from grid to global 3D position12:        $v\leftarrow T_{i}^{-1}v_{g}$13:        p← perspective project vertex *v*14:        **if** p∈ depth map $D_{i}$ and $D_{i}\left(p\right)>0$ **then**15:           $tsdf_{i}\leftarrow tsdf_{i-1}$16:           $w_{i}\leftarrow w_{i-1}$17:           **for** each $p_{k}$ in 3×3 area around *p* **do**18:               $\Delta u\leftarrow ||p-p_{k}\left|\right|$ with sub-pixel accuracy19:               θ← angle(*z*-axis, $n_{i}\left(p_{k}\right)$)20:               $\Delta z\leftarrow \left|\right|D_{i}\left(p\right)-D_{i}\left(p_{k}\right)\left|\right|$21:               **if** $D_{i}\left(p_{k}\right)>0$ and θ<$\theta_{max}$  **then**22:                   $\sigma_{Z}\leftarrow$ Look up from noise image (Figure 4) against $D_{i}\left(p_{k}\right)$ and θ23:                   $\sigma_{L}\leftarrow \left\{\begin{array}{cc} f_{x}\times 0.00041 & \mathrm{for}\;\theta \leq 60\\ f_{x}\times 0.0043 & \mathrm{for}\;\theta >60 \end{array}\right.$ (Figure 6)24:                   $sdf_{k}\leftarrow \left|\right|c_{i}-v\left|\right|-D_{i}\left(p_{k}\right)$25:                   **if** $sdf_{k}>-6\sigma_{z}$ and $\Delta z<3\sigma_{z}$ **then**26:                       $tsdf_{k}\leftarrow$ sgn($sdf_{k}$) $\sqrt{1-e^{-\frac{2{\mathrm{sdf}}_{k}^{2}}{\pi \sigma_{Z}^{2}}}}$27:                       $w_{k}\leftarrow \frac{\sigma_{z}\left(z_{\min}\right)}{\sigma_{Z}}\frac{z_{\min}^{2}}{D_{i}^{2}\left(p_{k}\right)}e^{-\frac{\Delta u^{2}}{2\sigma_{L}^{2}}-\frac{\Delta z^{2}}{2\sigma_{z}^{2}}}$28:                       $tsdf_{i}\leftarrow \frac{tsdf_{i}w_{i}+tsdf_{k}w_{k}}{w_{i}+w_{k}}$29:                       $w_{i}\leftarrow \min\left(\max\mathrm{weight},w_{i}+w_{k}\right)$30:                   **end if**31:               **end if**32:           **end for**33:        **end if**34:    **end while**35:**end for**

Algorithm S2, attached in the supplementary material, provides an efficient implementation of Algorithm 1. By skipping lateral noise, which requires iteration over the 3×3 neighborhood, this version significantly speeds up runtime (≈9 times) with a small performance drop (results in the Supplementary Materials).

**Figure 10:**
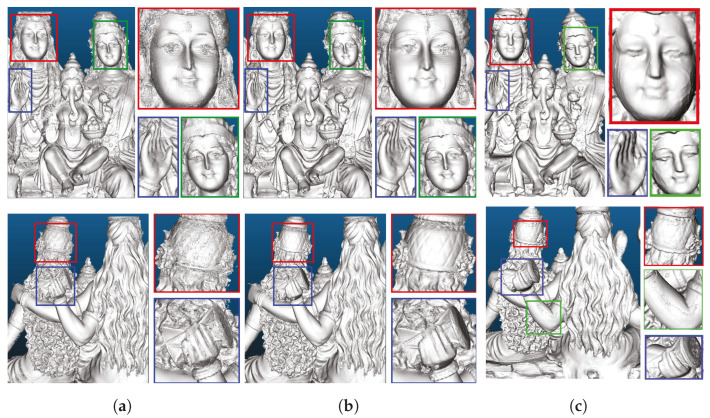
The qualitative results from the Shiva dataset, both top-front and bottom-back views, highlighting improved reconstruction quality (**b**), in the colored boxes, when noisy depth measurements are filtered using our noise model in the KinectFusion algorithm. This improvement is particularly noticeable compared with the reconstruction without depth filtering (**a**) and against the traditional reconstruction pipeline (**c**) discussed in Section 4.1.

Figure 10b illustrates the substantial improvement in reconstruction quality with the KinectFusion algorithm using our noise model compared with the results obtained without noise filtering (Figure 10a) and with a more traditional 3D reconstruction pipeline (Figure 10c), as explained in Section 4.1.

**Figure 11:**
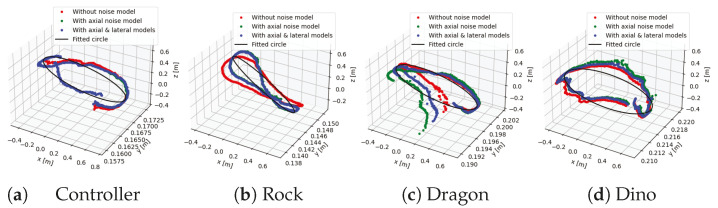
Comparison of trajectories for different objects with and without noise filtering. Results demonstrate superior trajectories with noise filtering (green and blue) as compared with those without noise (red). Fitted circle to the trajectory with axial and lateral noise models (blue). The axes are not of the same scale.

This depth filtering using our noise also improves tracking, as shown in Figure 11, where we observe a reduced drift in the trajectory when the depth is filtered. The decreased drift for the noisy depth filtering case is evident from the minimal difference between the start and end poses of the camera for 360° rotations, which is significantly large when noisy depth measurements are not filtered. The corresponding RMSE between the ground-truth poses and the trajectories obtained with and without noise filtering is presented in Table 1. The ground-truth poses were obtained from the calibrated rotating table and the robot arm setup, which were further refined by fitting a circular plane on the rotating axis.

Table 1 provides drift measurements (both distance and angle) as a quantitative comparison between different reconstruction conditions (without noise, with axial noise, and with axial and later noise models) for five datasets. The reconstruction with noise model gains significant improvements for Shiva, Controller and Rock, although it slightly underperforms reconstruction without noise models for Dragon and Dino. Most of the time, reconstruction with just axial noise performs close to that with both axial and lateral noise models, except for very smooth objects, and at a significant speed-up of approximately 9-fold.

Table 2 provides a quantitative evaluation of the Gripper mesh across four cases, reconstructed using ground-truth poses, compared with the ground-truth mesh. It emphasizes the critical role of depth map filtering using our empirically derived noise models in applications demanding submillimeter-level accuracy. The corresponding precision and recall plots across various distance thresholds are detailed in Figure S7 of the Supplementary Materials.

### 4.3. Improving Neural Implicit SLAM Using Our Noise Model

**Figure 12:**
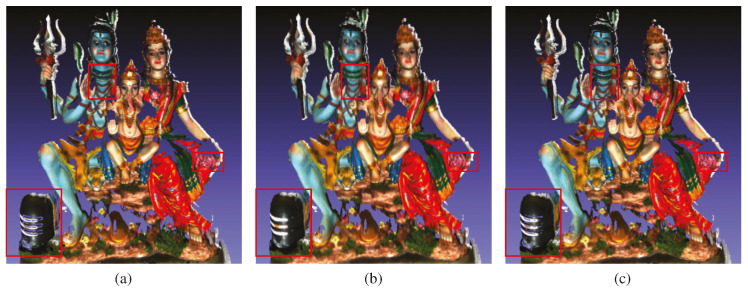
Qualitative results of integrating our noise model in Point-SLAM with the Shiva dataset. Highlighted boxes show improved reconstruction—Point-SLAM baseline (**a**), noisy depth filtering using our noise model (**b**), and depth loss term using our noise model (**c**). Please zoom in the red box for better visualization.

We are able to integrate our noise model in NeRF-based SLAM systems such as Point-SLAM [9], as shown in Figure 12, by incorporating uncertainty weighting and ground-truth depth refinement based on our noise model. The ground-truth depth refinement is performed by calculating the expected depth value from a neighborhood of 3×3 pixels using a Gaussian distribution that incorporates both axial and lateral noise. We use $\sigma_{z}\left(z_{\min}\right)/\sigma_{z}\left(z\right)$ as the uncertainty weight, where $\sigma_{z}\left(z_{\min}\right)$ represents the axial noise at the minimum depth value. Moreover, we incorporate this axial noise as a dynamic lower bound in the point search radius of Point-SLAM. This dynamic lower bound is critical for ensuring that the search for neighboring points spans a sufficient spatial range, especially for distant points.

Our noise modeling approach focuses primarily on high-resolution Zivid scans. Integrating our noise model significantly improves results when the reconstruction is performed on a very fine scale (0.2 mm or finer). This aligns well with traditional multi-view stereo and classical point-based reconstruction methods. Difficulties are encountered when applying our noise model to neural implicit approaches. When using Gaussian Splatting-based SLAM (such as Splat-TAM [43]), memory limitations restrict the resolution at which our noise model can be effectively utilized. When using NeRF-based SLAM (such as Point-SLAM [9]), we encounter computational performance bottlenecks. Incorporating high-resolution noise corrections efficiently into these methods will require further research.

## 5. Conclusions

In this paper, we empirically derive a noise model for the RGB-D Zivid sensor that quantifies the axial and lateral noise components as a function of distance and surface angle. Experimental results demonstrate quantitatively and qualitatively that our derived noise model leads to more accurate 3D reconstructions. Integrating this model into 3D reconstruction pipelines, through bilinear interpolation or fitted functions, yields significant improvements in both pose estimation and reconstruction accuracy. Therefore, the use of our method can be recommended in advanced manufacturing and other applications that require both high resolution and reduced measurement uncertainty. Additionally, we provide a publicly available dataset of high-resolution RGB-D scans to facilitate future applied research.

A limitation of our current approach is that it is based on scanning calibration targets to accurately estimate sensor noise characteristics. A promising direction for future research is the development of self-supervised or data-driven methods that can infer noise characteristics directly from captured scenes without requiring explicit calibration setups. Another limitation is that, while we have empirically modeled the impact of environmental factors such as temperature and background lighting on sensor noise, we have not incorporated these effects into our reconstruction pipeline. Future work can focus on dynamically integrating these models to improve the robustness of reconstruction under varying environmental conditions. Additionally, although our noise model has demonstrated an improvement in 3D reconstruction, we have not systematically studied how different reconstruction paradigms, such as traditional multi-view stereo versus implicit neural representations, are affected by noise. A potential research direction is to analyze how our noise model can be uniformly applied across these diverse approaches to ensure both consistency and robustness in reconstruction. Finally, our current noise model focuses primarily on estimating the standard deviation of noise. However, sensor noise can also introduce systematic biases that affect the accuracy of the reconstruction. Future research should explore methods to estimate and compensate for these bias components in RGB-D sensor data, enabling more precise 3D reconstructions.

## Figures and Tables

**Table 1:** Drift values for translation and rotation (m, degrees) of first and last estimated camera poses of different objects.

Objects	Without Noise	Axial Noise	Axial-Lateral Noise
Shiva	(0.00613, 0.42107)	(0.00184, 0.12915)	(**0.00178, 0.12375**)
Controller	(1.13467, 33.417)	(1.08114, 48.397)	(**0.00579, 0.58854**)
Rock	(0.00240, 0.31135)	(0.00086, 0.13408)	(**0.00080, 0.10139**)
Dragon	(**0.00250, 0.41372**)	(0.01116, 1.7199)	(0.00820, 1.2446)
Dino	(**0.01085, 1.159**)	(0.01121, 1.1883)	(0.01131, 1.2041)

**Table 2:** Comparison of Gripper mesh with a ground-truth mesh in terms of precision (Prec.), recall (Rec.), and F-score (F) at a distance threshold of 0.1 mm.

Baseline			Without Noise			Axial Noise			Axial-Lateral Noise		
Prec.↑	Rec.↑	F↑	Prec.↑	Rec.↑	F↑	Prec.↑	Rec.↑	F↑	Prec.↑	Rec.↑	F↑
0.154	0.435	0.228	0.361	0.479	0.412	0.382	**0.485**	0.427	**0.385**	0.482	**0.428**

## Data Availability

Data are contained within the article and Supplementary Materials.

## Supplementary Materials

The following supporting information can be downloaded at: https://www.mdpi.com/article/10.3390/s25030950/s1, Figure S1: Comparison between 3D reconstruction obtained using a full noise model incorporating both axial and lateral noise components (left), axial noise using Algorithm 1 of the main text (middle), and Algorithm S1 (right). We can see slight compromise in the quality with the later (right) but at a roughly nine fold improved speed; Figure S2: Comparison of reconstruction quality using ground truth camera poses, without noise (left), with axial noise only (middle), and with both axial and lateral noise (right). Our noise model integrated with TSDF demonstrates superior reconstruction quality as shown in zoomed in images at the bottom of each figure. When comparing the effectiveness of noise filtering using just axial noise versus utilizing both axial and lateral noise, we observe that incorporating both components yields higher resolution for fine details in the reconstructed depth data; Figure S3: Comparison of the quality of reconstructions (top) and trajectories (bottom) with unfiltered depth maps on the left and filtered results with axial noise in the middle and both axial and lateral noise on the right. Depth map filtering using our noise model improves both the reconstruction quality and camera pose tracking. Note that the axes of trajectories are not of the same scale; Figure S4: Reconstruction results (top to bottom) for Controller, Rock, Dragon, and Dino. From left to right we have without noise filtering, with axial noise filtering, and with both axial and lateral noise filtering. On these relatively smooth objects, the difference in reconstruction quality between filtered and unfiltered versions is minimal. This is attributed to the lack of high resolution geometric features in these objects which our noise model significantly enhances; Figure S5: Poor quality of the gripper’s mesh using the Kinect noise model compared to the Zivid noise model in Figure S10; Figure S6: Poor quality of the gripper’s mesh using the Gaussian filtering compared to the Zivid noise model in Figure S10; Figure S7: Precision and recall metrics across different distance thresholds for four meshes: (a) baseline method, (b) without noise filtering, (c) with axial noise filtering, and (d) with both axial and lateral noise filtering; Figure S8: Precision and Recall meshes along with their histograms for baseline method; Figure S9: Precision and Recall meshes along with their histograms without depth filtering; Figure S10:Precision and Recall meshes along with their histograms for axial noise filtering case; Figure S11: Precision and Recall meshes along with their histograms for axial and lateral noise filtering case; Table S1: Coefficients for $f_{1}\left(z,\theta \right)$; Table S2: Coefficients for $f_{2}\left(z,\theta \right)$; Table S3: Axial noise values corresponding to Figure 4a of the main text; Algorithm S1: TSDF integration with axial noise only.
