# Supplementary material for: Improving 3D Reconstruction Through RGB-D Sensor Noise Modeling
Source: Sensors (Basel). 2025 Feb 5;25(3):950. doi: 10.3390/s25030950 (PMC11819956; doi:10.3390/s25030950)
Supplement: Supplementary file 1 [file sensors-25-00950-s001.zip › sensors-3362180-supplementary.pdf]

sensors

MDPI

Article

Supplementary Material for "Improving 3D Reconstruction Through RGB-D Sensor Noise Modeling"

Fahira Afzal Maken [1,*,†], Sundaram Muthu [1,†], Chuong Nguyen [1], Changming Sun [1], Jinguang Tong [1,2], Shan Wang [1,2], Russell Tsuchida [1], David Howard [1], Simon Dunstall [1], Lars Petersson [1]

[1] Data61, Commonwealth Scientific and Industrial Research Organisation (CSIRO), Canberra, ACT, 2601, Australia; sundaram.muthu@data61.csiro.au (S.M.); chuong.nguyen@data61.csiro.au (C.N.); changming.sun@data61.csiro.au (C.S.); jinguang.tong@anu.edu.au (J.T.); shan.wang@anu.edu.au (S.W.); russell.tsuchida@data61.csiro.au (R.T.); david.howard@data61.csiro.au (D.H.); simon.dunstall@data61.csiro.au (S.D.); lars.petersson@data61.csiro.au (L.P.);

[2] School of Computing, Australian National University (ANU),Canberra, ACT 2601, Australia

* Correspondence: fahira.afzalmaken@data61.csiro.au

† These authors contributed equally to this work.

check for updates

Academic Editor: Miguel Oliveira

Received: 3 December 2024

Revised: 20 January 2025

Accepted: 21 January 2025

Published: 5 February 2025

Citation: Afzal Maken, F.; Muthu, S.; Nguyen, C.; Sun, C.; Tong, J.; Wang, S.; Tsuchida, R.; Howard, D.; Dunstall, S.; Petersson, L. Improving 3D Reconstruction Through RGB-D Sensor Noise Modeling. *Sensors* **2025**, *25*, 950. https://doi.org/10.3390/s25030950

Copyright: © 2025 by the authors. Licensee MDPI, Basel, Switzerland. This article is an open access article distributed under the terms and conditions of the Creative Commons Attribution (CC BY) license (https://creativecommons.org/licenses/by/4.0/).

1. Noise Modelling and KinectFusion

Supposing a $2D$ pixel coordinate on the depth map is denoted as $u = (x, y)$. $D_i(u)$ is the depth value at pixel u retrieved at the ith frame. With an intrinsic calibration matrix K, a 3D vertex of the pixel u is $v_i(u) = D_i(u)K^{-1}[u, 1]$. D_i therefore results in a single vertex map V_i. With the camera pose of the ith frame $T_i = [R_i, c_i]$. The vertex position is expressed in global coordinates as $v_{g_i} = T_i \cdot v_i$.

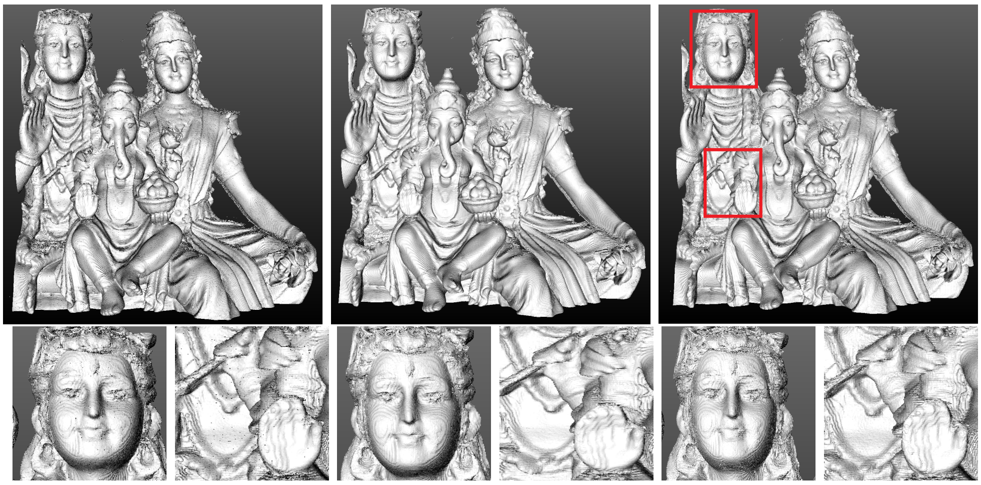

Figure S1. Comparison between 3D reconstruction obtained using a full noise model incorporating both axial and lateral noise components (left), axial noise using Algorithm 1 of the main text (middle), and Algorithm S1 (right). We can see slight compromise in the quality with the later (right) but at a roughly nine fold improved speed.

Algorithm S1 provides an efficient implementation of Algorithm 1 of the main text. By skipping lateral noise, which requires iteration over the 3×3 neighborhood, this version significantly speeds up runtime ($\approx$ 9 times) with a small performance drop as shown in Fig. S1.

Algorithm S1 TSDF integration with axial noise only

Depth map D_i for frame i
Camera pose T_i consisting of rotation matrix R_i and translation vector c_i
Noise models: σ_Z, σ_L
Angle threshold θ_{max}
Output:
Updated TSDF $tsdf_i$ for the current frame
for each voxel g on x-y slice of volume in parallel **do**
  while sweep along z-axis of volume **do**
    $v_g \leftarrow$ convert g from grid to global 3D position
    $v \leftarrow T_i^{-1} v_g$
    $p \leftarrow$ perspective project vertex v
    if $p \in$ depth map D_i and $D_i(p) > 0$ **then**
      $tsdf_i \leftarrow tsdf_{i-1}$
      $w_i \leftarrow w_{i-1}$
      $\theta \leftarrow$ angle(z-axis, $n_i(p_k)$)
      if $D_i(p_k) > 0$ and $\theta <$ angle threshold **then**
        $\sigma_Z \leftarrow$ Look up from noise image (Fig. 4 in the main text) against $D_i(p_k)$ and θ
        $sdf_k \leftarrow ||c_i - v|| - D_i(p_k)$
        if $sdf_k > -6\sigma_z$ **then**
          $tsdf_k \leftarrow \text{sgn}(sdf_k)\sqrt{1 - e^{-\frac{2\text{sdf}_k^2}{\pi\sigma_Z^2}}}$
          $w_k \leftarrow \frac{\sigma_z(z_{\text{min}})}{\sigma_Z}\frac{z_{\text{min}}^2}{D_i^2(p_k)}$
          $tsdf_i \leftarrow \frac{tsdf_i w_i + tsdf_k w_k}{w_i + w_k}$
          $w_i \leftarrow \min(\max\text{weight}, w_i + w_k)$
        end if
      end if
    end if
  end while
end for

2. Axial Noise Values and Corresponding Coefficient Values for Surface Fitting Models

The coefficient values for surface fitting models for $f_1(z,\theta) = \sum_{i=0}^{6}\sum_{j=0}^{6} a_{ij} z^i \theta^j$ and $f_2(z,\theta) = Se^{Az^2+Bz\theta+C\theta^2+Dz+E\theta} + az^2 + bz\theta + c\theta^2 + dz + e\theta + f$ are provided in Table S1 and S2. Axial noise value corresponding to Figure 4 (a) of the main text are provided in Table S3.

Table S1. Coefficients for $f_1(z,\theta)$

a_{00}	a_{01}	a_{02}	a_{03}	a_{04}	a_{05}	a_{10}	a_{11}
1.779×10^{11}	-50.05	-17.47	201.09	121.38	42.32	-444.45	-318.67
a_{12}	a_{13}	a_{14}	a_{15}	a_{20}	a_{21}	a_{22}	a_{23}
-289.94	-8.95	582.32	429.92	598.98	258.15	-86.45	-449.62
a_{24}	a_{25}	a_{30}	a_{31}	a_{32}	a_{33}	a_{34}	a_{35}
-328.77	-525.59	-513.95	-18.44	119.08	188.73	139.91	195.19
a_{40}	a_{41}	a_{42}	a_{43}	a_{44}	a_{45}	a_{50}	a_{51}
350.57	134.07	-66.96	-64.58	-33.10	-25.99	-23.00	-75.36
a_{52}	a_{53}	a_{54}	a_{55}	-	-	-	-
-61.01	0.353	22.36	13.20	-	-	-	-

Table S2. Coefficients for $f_2(z, \theta)$

Parameter	Value
S	5.921×10^{-25}
A	-6.084
B	-31.842
C	1.878
D	54.610
E	35.759
a	0.768
b	0.119
c	0.045
d	-0.752
e	-0.070
f	0.224

Table S3. Axial noise values corresponding to Figure 4 (a) of the main text.

z(mm)	$\theta°$	σ_Z (mm)	z(mm)	$\theta°$	σ_Z (mm)	z(mm)	$\theta°$	σ_Z (mm)
1070	0	0.2776	1045	0	0.2602	1020	0	0.2392
995	0	0.2277	970	0	0.1919	945	0	0.1770
920	0	0.1651	895	0	0.1514	870	0	0.1274
845	0	0.1168	820	0	0.1071	795	0	0.0985
770	0	0.0837	745	0	0.0739	720	0	0.0672
695	0	0.0609	670	0	0.0517	645	0	0.0462
620	0	0.0428	595	0	0.0398	570	0	0.0362
545	0	0.0363	520	0	0.0382	495	0	0.0417
470	0	0.0438	445	0	0.0497	420	0	0.0557
395	0	0.0616	370	0	0.0675			
1070	10	0.3247	1045	10	0.2939	1020	10	0.2711
995	10	0.2481	970	10	0.2256	945	10	0.2082
920	10	0.1924	895	10	0.1751	870	10	0.1599
845	10	0.1463	820	10	0.1347	795	10	0.1220
770	10	0.1116	745	10	0.1017	720	10	0.0922
695	10	0.0833	670	10	0.0759	645	10	0.0685
620	10	0.0624	595	10	0.0564	570	10	0.0517
545	10	0.0472	520	10	0.0431	495	10	0.0395
470	10	0.0374	445	10	0.0359	420	10	0.0355
395	10	0.0372	370	10	0.0415			
1070	20	0.3480	1045	20	0.3152	1020	20	0.2843
995	20	0.2608	970	20	0.2397	945	20	0.2202
920	20	0.2008	895	20	0.1829	870	20	0.1695
845	20	0.1531	820	20	0.1415	795	20	0.1279
770	20	0.1179	745	20	0.1058	720	20	0.0959
695	20	0.0866	670	20	0.0783	645	20	0.0710
620	20	0.0642	595	20	0.0583	570	20	0.0532
545	20	0.0486	520	20	0.0443	495	20	0.0409
470	20	0.0386	445	20	0.0373	420	20	0.0375
395	20	0.0391	370	20	0.0438			
1070	30	0.3891	1045	30	0.3417	1020	30	0.3119
995	30	0.2847	970	30	0.2579	945	30	0.2382
920	30	0.2151	895	30	0.1964	870	30	0.1791
845	30	0.1638	820	30	0.1502	795	30	0.1355
770	30	0.1226	745	30	0.1124	720	30	0.1024
695	30	0.0919	670	30	0.0831	645	30	0.0754
620	30	0.0683	595	30	0.0622	570	30	0.0569

545	30	0.0513	520	30	0.0484	495	30	0.0447
470	30	0.0426	445	30	0.0413	420	30	0.0411
395	30	0.0441	370	30	0.0506			
1070	40	0.4696	1045	40	0.4021	1020	40	0.3697
995	40	0.3279	970	40	0.2895	945	40	0.2633
920	40	0.2367	895	40	0.2148	870	40	0.1977
845	40	0.1792	820	40	0.1630	795	40	0.1502
770	40	0.1347	745	40	0.1221	720	40	0.1107
695	40	0.1016	670	40	0.0912	645	40	0.0829
620	40	0.0751	595	40	0.0687	570	40	0.0624
545	40	0.0580	520	40	0.0540	495	40	0.0505
470	40	0.0488	445	40	0.0482	420	40	0.0486
395	40	0.0513	370	40	0.0569			
1070	50	0.6294	1045	50	0.5534	1020	50	0.4889
995	50	0.4264	970	50	0.3704	945	50	0.3296
920	50	0.2920	895	50	0.2605	870	50	0.2303
845	50	0.2062	820	50	0.1882	795	50	0.1686
770	50	0.1533	745	50	0.1389	720	50	0.1284
695	50	0.1157	670	50	0.1041	645	50	0.0961
620	50	0.0862	595	50	0.0800	570	50	0.0734
545	50	0.0684	520	50	0.0643	495	50	0.0619
470	50	0.0601	445	50	0.0602	420	50	0.0611
395	50	0.0648	370	50	0.0695			
1070	60	0.9992	1045	60	0.8757	1020	60	0.7502
995	60	0.6584	970	60	0.5652	945	60	0.4828
920	60	0.4269	895	60	0.3711	870	60	0.3125
845	60	0.2709	820	60	0.2394	795	60	0.2175
770	60	0.1872	745	60	0.1704	720	60	0.1556
695	60	0.1375	670	60	0.1234	645	60	0.1121
620	60	0.1025	595	60	0.0943	570	60	0.0860
545	60	0.0801	520	60	0.0762	495	60	0.0720
470	60	0.0701	445	60	0.0703	420	60	0.0725
395	60	0.0771	370	60	0.0827			
1070	70	2.1527	1045	70	2.0593	1020	70	1.8498
995	70	1.7376	970	70	1.5851	945	70	1.4280
920	70	1.3034	895	70	1.1879	870	70	1.0540
845	70	0.9444	820	70	0.8390	795	70	0.7336
770	70	0.6378	745	70	0.5544	720	70	0.4813
695	70	0.4127	670	70	0.3526	645	70	0.3126
620	70	0.2714	595	70	0.2392	570	70	0.2086
545	70	0.1856	520	70	0.1677	495	70	0.1496
470	70	0.1411	445	70	0.1394	420	70	0.1324
395	70	0.1311	370	70	0.1265			

3. Ablation Study

We investigate the impact of various components of KinectFusion on the quality of 3D reconstruction. Initially, we utilize ground truth poses to reconstruct the object, aiming to see the effect of noise filtering with our noise model, comparing it to quality of reconstruction without any noise filtering.

Figure S2 provides a comparison of reconstruction quality using different noise filtering models using fixed ground truth camera poses. Despite the camera poses being fixed at ground truth values, the reconstruction quality is notably superior when integrating our noise model with TSDF (middle and right). This improvement is evident from the zoomed-in images at the bottom of each figure, highlighting finer details captured in the reconstruction. Additionally, a further examination of the

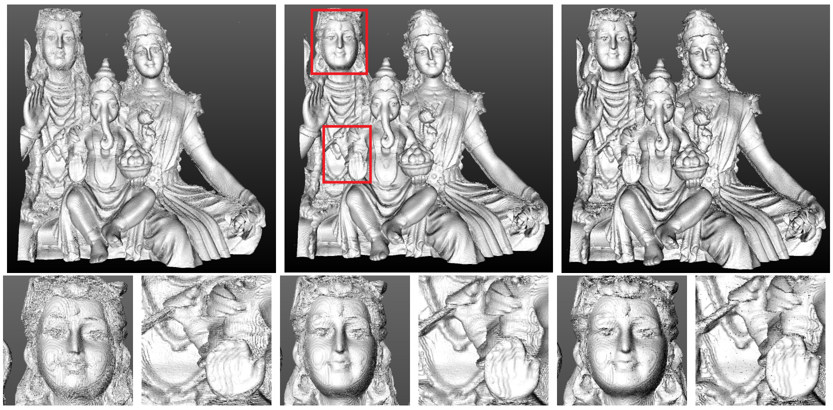

Figure S2. Comparison of reconstruction quality using ground truth camera poses, without noise (left), with axial noise only (middle), and with both axial and lateral noise (right). Our noise model integrated with TSDF demonstrates superior reconstruction quality as shown in zoomed in images at the bottom of each figure. When comparing the effectiveness of noise filtering using just axial noise versus utilizing both axial and lateral noise, we observe that incorporating both components yields higher resolution for fine details in the reconstructed depth data.

effectiveness of noise filtering reveals that incorporating both axial and lateral noise components results in higher resolution compared to just axial noise filtering.

Next, we run full KinectFusion pipeline with ICP, providing camera poses, with and without noise filtering. We then examine the quality of trajectory obtained without noise filtering, with axial noise filtering alone, and with both axial and lateral noise filtering. Figure S3 presents a comparison between trajectories and reconstruction quality with and without depth filtering using our noise model. Notably, trajectories exhibit significant drift and reconstruction quality suffers when depths are left unfiltered. When tracked without ground truth poses, the quality with depth filtering remains consistent. This is attributed to the high accuracy of tracking, which closely aligns with the ground truth poses, when depth maps are filtered.

4. Results - Other Datasets

Reconstruction results, as shown in Fig. S4, on additional datasets reveal insights into the impact of noise filtering. Smooth objects with limited high-resolution features show little difference between the reconstructions with and without noise filtering attributed to the absence of intricate geometry that would otherwise reveal compromised quality without filtering. Conversely, objects characterized by well-defined geometries demonstrate notable improvement with noise filtering as evident in Figs. S2 and S3. This suggests that our filtering methodology enhances reconstruction quality, particularly for objects with high definition geometries. Corresponding trajectories for additional dataset are shown in Fig 11 of the main text.

5. Testing Kinect Noise Model and Gaussian Filtering Effectiveness on Zivid Data

Using the Kinect noise model leads to poor quality in the gripper mesh, as shown in Fig. S5, highlighting the need for modeling noise characteristics of the Zivid sensor. The accuracy and resolution of the two sensors differ significantly; the Zivid sensor achieves sub-millimeter accuracy and high resolution, far surpassing the Kinect. Additionally, the Kinect has a working distance of about 0.5 to 4.5 meters, while the Zivid works best at around 0.3 to 1.5 meters. This difference highlights the limitations of using the Kinect noise model for Zivid data. Applying this model to Zivid data can severely filter out accurate depth measurements, as it is designed for lower resolution and less accurate depth data. Similarly, applying generalized Gaussian filtering can either oversmooth the data or introduce noise into the resulting meshes. This is due to the filter's tendency to reduce detail excessively while failing to adequately manage variations in depth data. Consequently, important features may be lost, leading to a lack of fidelity in the mesh representation. Depth filtering using a Gaussian filter with a sigma of 0.6mm, which is twice the depth resolution of the Zivid sensor, results in a significant loss of high-resolution depth details on the surfaces of

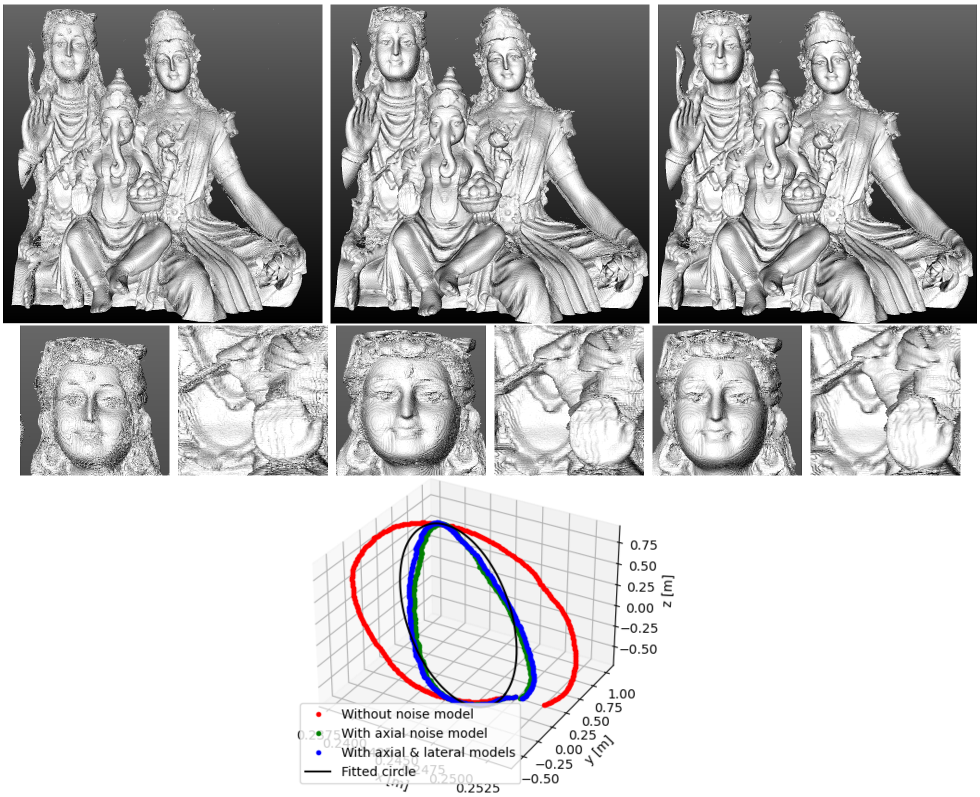

Figure S3. Comparison of the quality of reconstructions (top) and trajectories (bottom) with unfiltered depth maps on the left and filtered results with axial noise in the middle and both axial and lateral noise on the right. Depth map filtering using our noise model improves both the reconstruction quality and camera pose tracking. Note that the axes of trajectories are not of the same scale.

the gripper, while still producing a noisy mesh. This is evident in Fig. S6 when comparing the filtered output with the zivid's noise model presented in Fig. S10.

6. Quantitative Mesh Evaluation Against Ground Truth

Given that the ground truth mesh is available only for the gripper, in this section, we quantitatively evaluate the mesh quality across four scenarios: (a) baseline method (b) without any depth filtering, (c) with depth filtering using axial noise only, and (d) with both axial and lateral noise filtering. We assess these cases based on precision, recall, and F-score metrics. The results are visualized in Fig. S7, where precision and recall metrics are plotted against various distance threshold values.

The evaluation of mesh quality in terms of precision and recall yields several insights from the data plotted in Fig. S7. At a fine distance threshold of 0.0001 meters, meshes benefit significantly from axial and lateral noise depth filtering techniques, outperforming scenarios without depth filtering and even surpassing the baseline method using traditional ICP alignment and generic point cloud filtering methods. These enhancements are particularly crucial in digital manufacturing, where achieving high accuracy at finer scales ensures quality and precision in production processes. However, as the distance threshold increases, precision and recall values for methods involving axial noise, axial noise with lateral noise, and no depth filtering tend to converge. This suggests that while initial improvements are noticeable at finer resolutions, the distinctions diminish with broader thresholds. Overall, these findings underscore the efficacy of noisy depth filtering using axial and lateral noise models in improving reconstruction accuracy, especially where precision at finer scales is essential for manufacturing operations. The corresponding precision and recall meshes along with the histograms for all four methods are shown in Fig. S8 to S11 showing improved precision and recall with our noise model filtering.

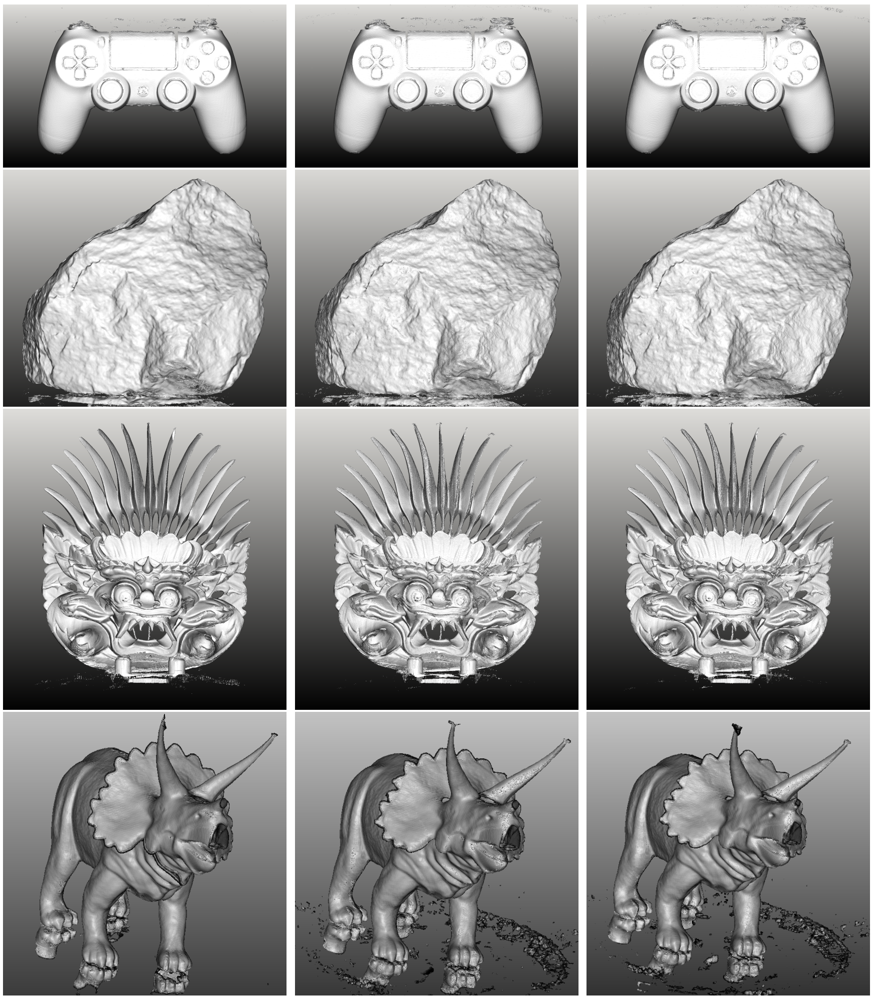

Figure S4. Reconstruction results (top to bottom) for Controller, Rock, Dragon, and Dino. From left to right we have without noise filtering, with axial noise filtering, and with both axial and lateral noise filtering. On these relatively smooth objects, the difference in reconstruction quality between filtered and unfiltered versions is minimal. This is attributed to the lack of high resolution geometric features in these objects which our noise model significantly enhances.

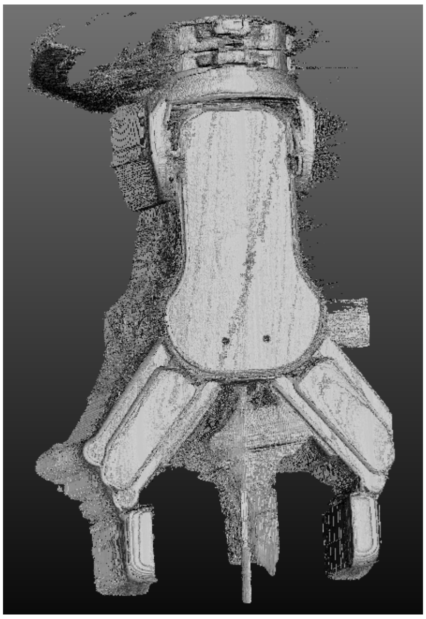

(**a**) With Kinect's axial noise model filtering

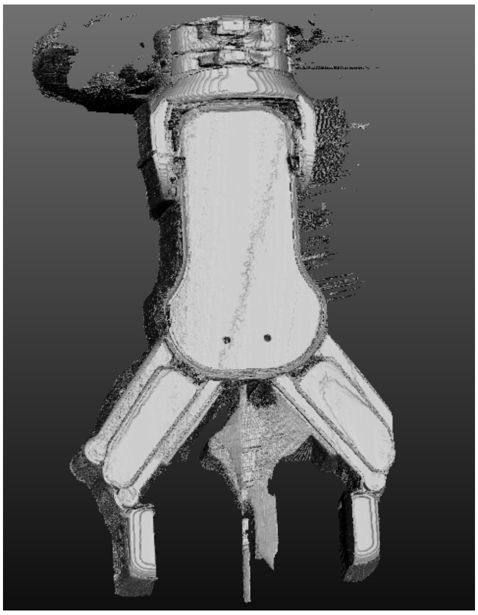

(**b**) With Kinect's axial and lateral noise model filtering

Figure S5. Poor quality of the gripper's mesh using the Kinect noise model compared to the Zivid noise model in Figure S10.

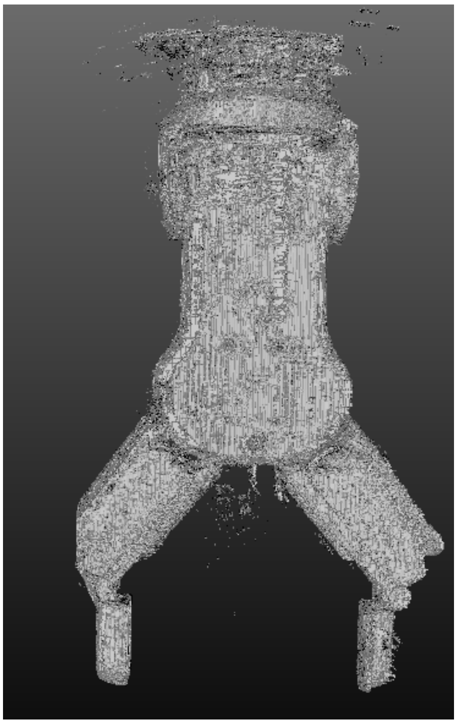

(**a**) Gaussian depth filtering with sigma=0 6mm-front side

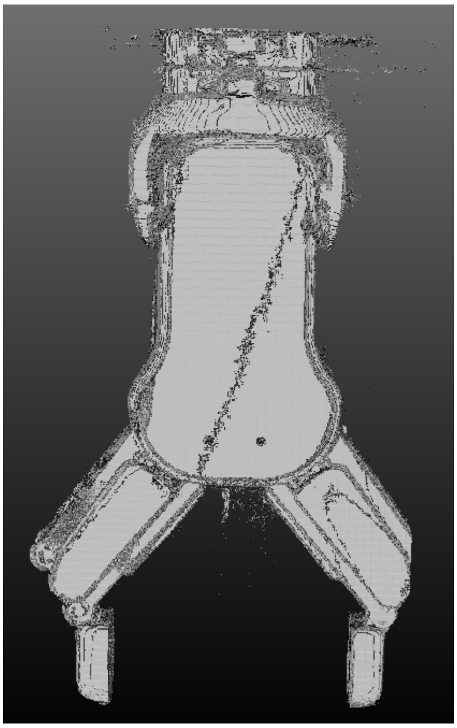

(**b**) Gaussian depth filtering with sigma=0 6mm-back side

Figure S6. Poor quality of the gripper's mesh using the Gaussian filtering compared to the Zivid noise model in Figure S10.

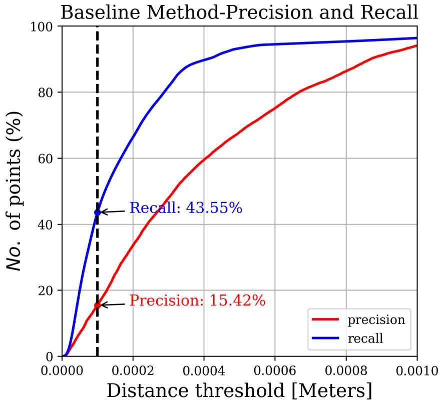

(**a**) Baseline Method

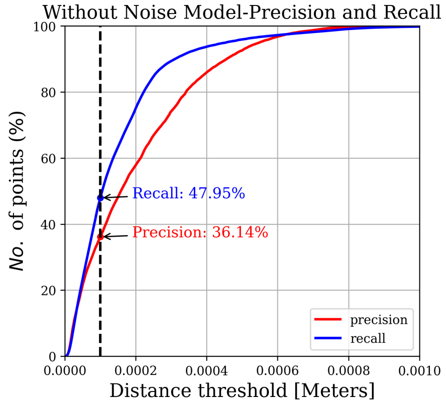

(**b**) Without noise filtering

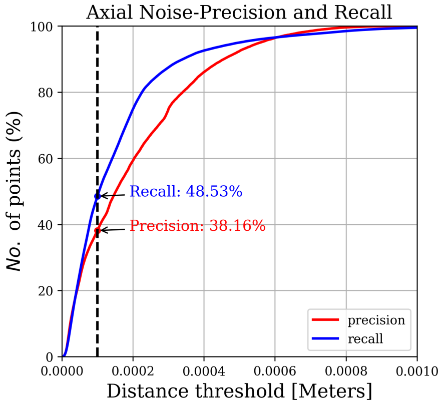

(**c**) With Axial noise filtering

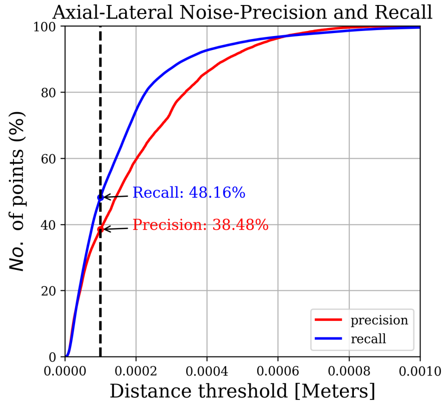

(**d**) With axial-lateral noise filtering

Figure S7. Precision and recall metrics across different distance thresholds for four meshes: (a) baseline method, (b) without noise filtering, (c) with axial noise filtering, and (d) with both axial and lateral noise filtering.

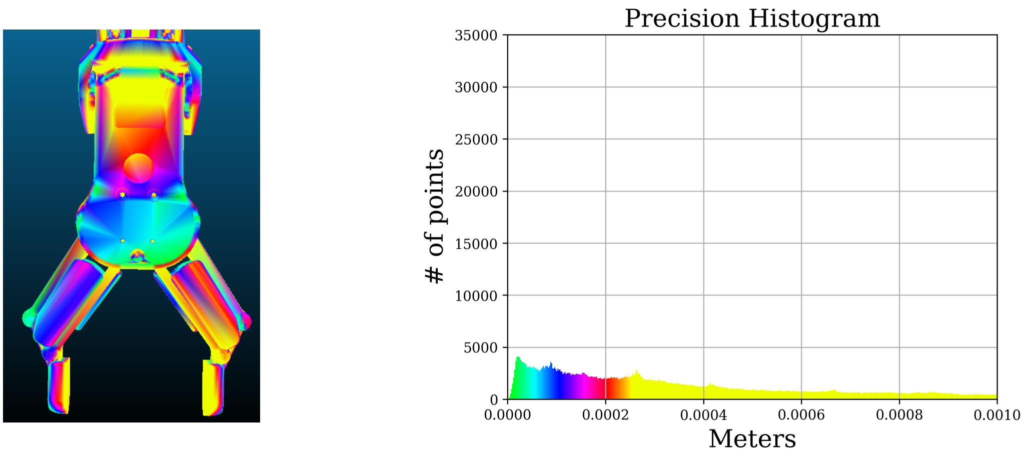

(**a**) Baseline Method - Precision mesh and histogram

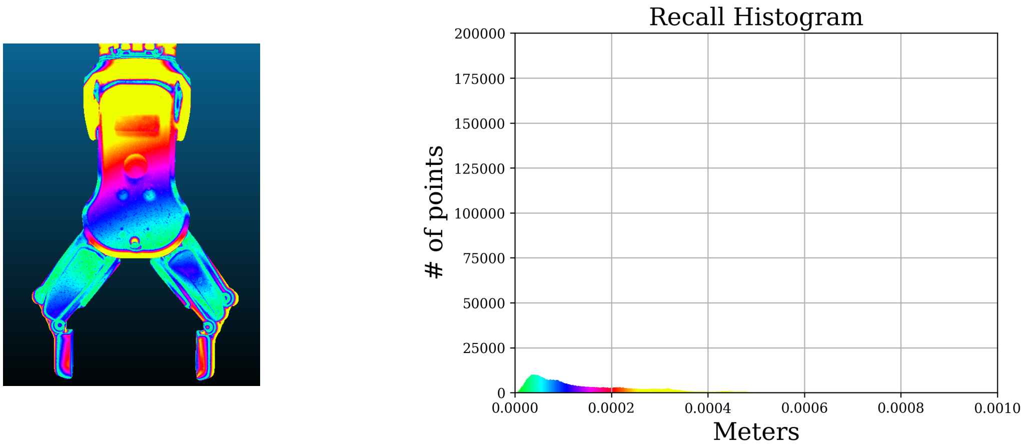

(**b**) Baseline Method - Recall mesh and histogram

Figure S8. Precision and Recall meshes along with their histograms for baseline method.

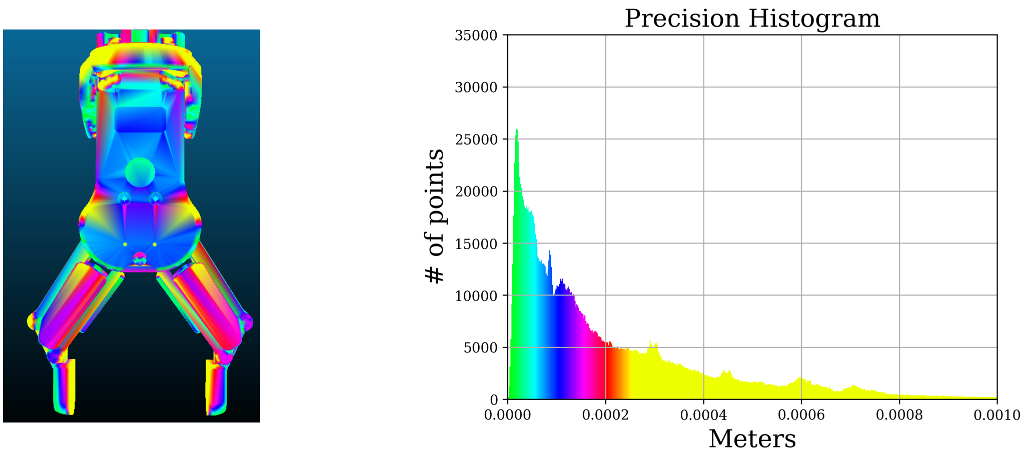

(**a**) Without Noise - Precision mesh and histogram

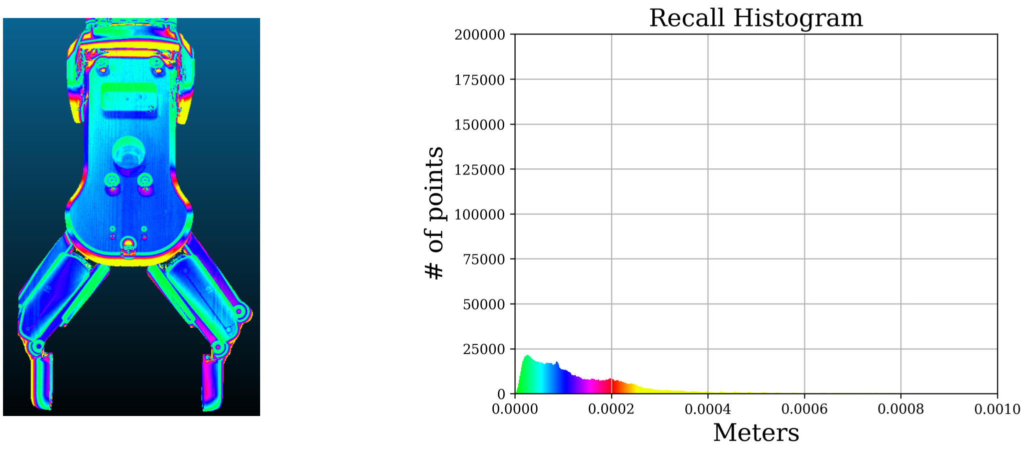

(**b**) Without Noise - Recall mesh and histogram

Figure S9. Precision and Recall meshes along with their histograms without depth filtering.

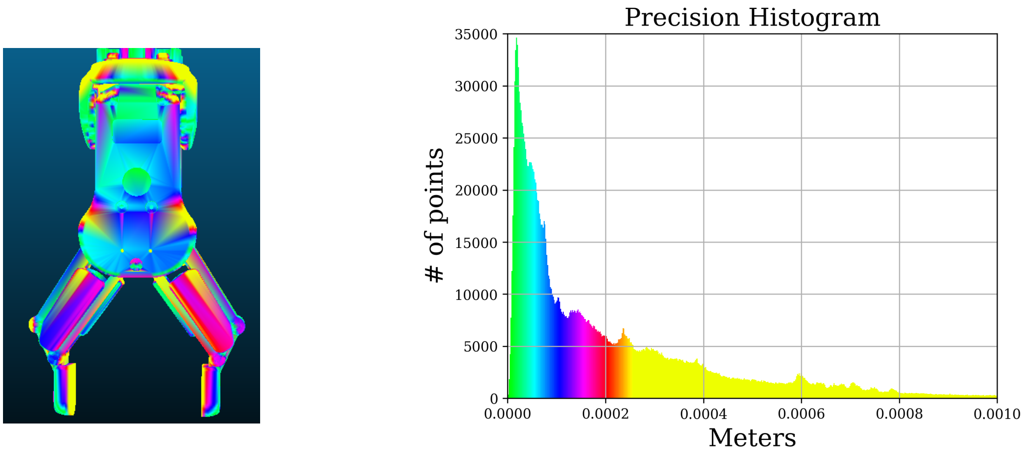

(**a**) With Axial Noise - Precision mesh and histogram

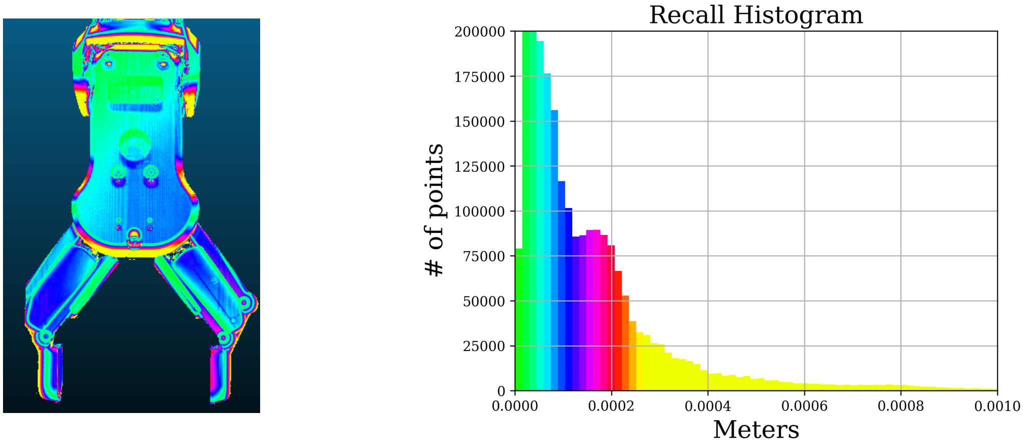

(**b**) With Axial Noise - Recall mesh and histogram

Figure S10. Precision and Recall meshes along with their histograms for axial noise filtering case.

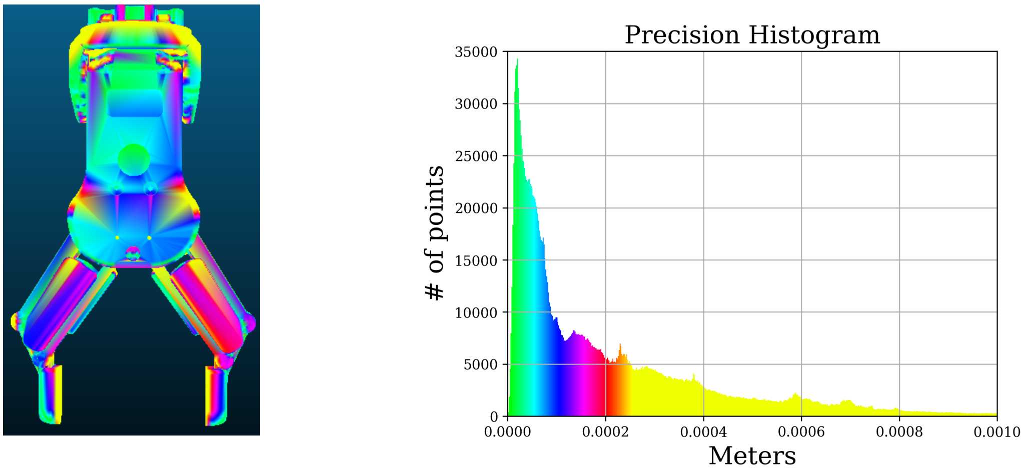

(**a**) With Axial-lateral Noise - Precision mesh and histogram

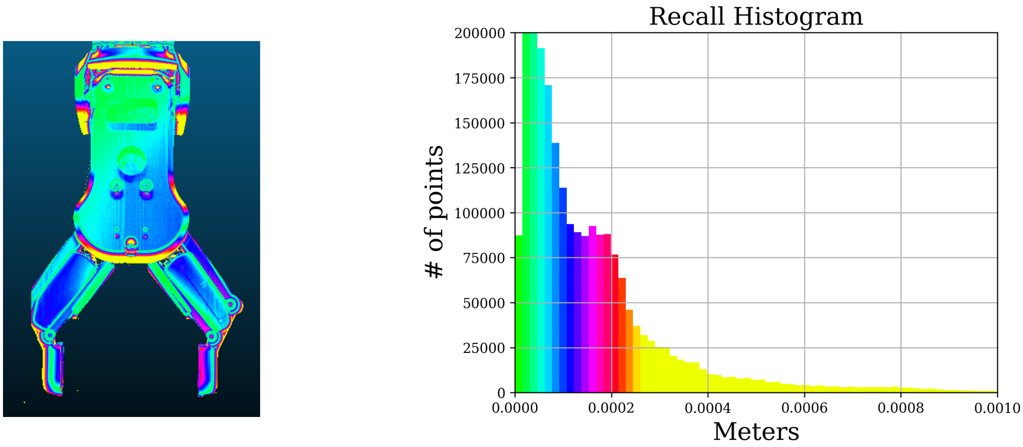

(**b**) With Axial-lateral Noise - Recall mesh and histogram

Figure S11. Precision and Recall meshes along with their histograms for axial and lateral noise filtering case.
